# A Case of Cytokine Release Syndrome (CRS) Related to Treatment With Lenvatinib Plus Pembrolizumab for Metastatic Clear Cell Renal Cell Carcinoma

**DOI:** 10.1002/iju5.70228

**Published:** 2026-08-07

**Authors:** Kei Nagase, Hiroaki Kakinoki, Kazuki Nakamura, Yukako Yamaguchi, Shuhei Kusano, Yuka Kakinoki, Maki Kawasaki, Kazuma Udo, Shohei Tobu, Mitsuru Noguchi

**Affiliations:** ^1^ Department of Urology Imari‐Arita Kyoritsu Hospital Saga Japan; ^2^ Department of Urology, Faculty of Medicine Saga University Saga Japan; ^3^ Department of Urology Youmeikai Obase Hospital Fukuoka Japan; ^4^ Department of Urology Kumamoto Kenhoku Hospital Kumamoto Japan

**Keywords:** cytokine release syndrome (CRS), lenvatinib, pembrolizumab, renal cell carcinoma

## Abstract

**Introduction:**

Cytokine release syndrome (CRS) is a systemic inflammatory response triggered by various factors, including infection and immunotherapy. Due to its nonspecific presentation, distinguishing CRS from other life‐threatening conditions remains challenging.

**Case Presentation:**

A 79‐year‐old male receiving lenvatinib plus pembrolizumab for metastatic renal cell carcinoma presented with high‐grade fever, altered mental status, and respiratory failure on day 4 of treatment. After excluding infectious etiologies and endocrine dysfunction, CRS was suspected and confirmed by elevated interleukin‐6 levels. Lenvatinib and pembrolizumab were discontinued, and the patient was treated with tocilizumab and dexamethasone. Clinical improvement was achieved by day 7.

**Conclusion:**

CRS should be considered a potential cause of unexplained shock in patients receiving immune checkpoint inhibitors. Early diagnostic consideration is vital for timely intervention.

## Keynote Message

One of the recently recognized immune‐related adverse events is CRS. Considering CRS as a potential cause of unexplained shock in patients receiving immunotherapy is essential.

## 1 Introduction

The combination of lenvatinib, a multitarget tyrosine kinase inhibitor (TKI), and pembrolizumab, an anti‐programmed cell death‐1 (PD‐1) antibody, is a standard first‐line treatment for advanced renal cell carcinoma. As clinical trials have demonstrated significant survival benefits, immune checkpoint inhibitors (ICIs) like pembrolizumab are increasingly used in oncological practice [1]. However, cytokine release syndrome (CRS) has recently emerged as a critical immune‐related adverse event (irAE). Characterized by a systemic inflammatory response, CRS is often difficult to distinguish from other life‐threatening conditions [2]. Here, we report a case of CRS secondary to pembrolizumab treatment for metastatic renal cell carcinoma.

## 2 Case Presentation

**FIGURE 1:**
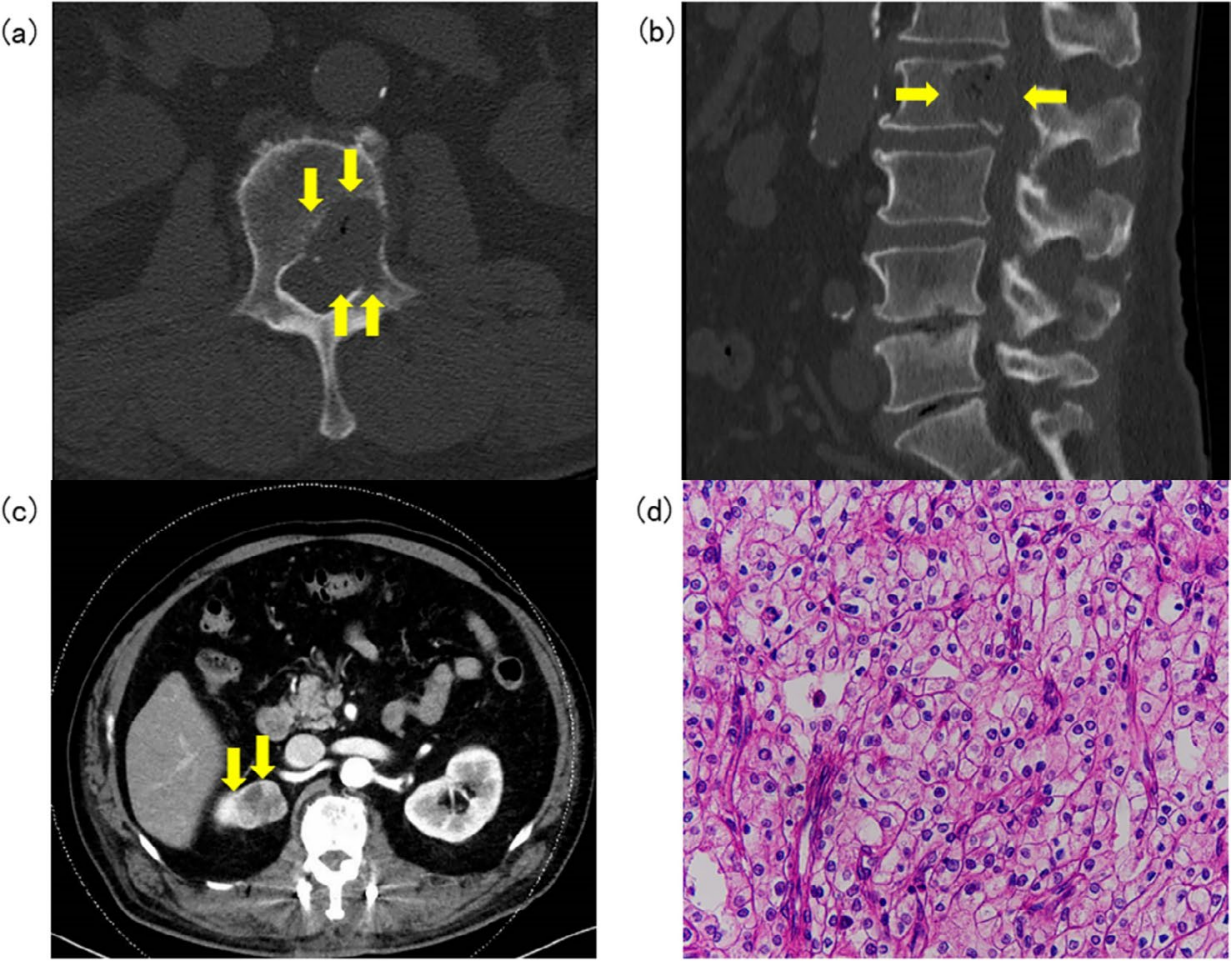
Initial diagnostic imaging of the patient. CT revealed bone mass in the L2 vertebra. (a) Coronal plane. (b) Sagittal plane. (c) Contrast‐enhanced CT revealed a mass in the upper pole of the right kidney. (d) Pathological examination of the resected tumor revealed clear cell renal cell carcinoma.

**FIGURE 2:**
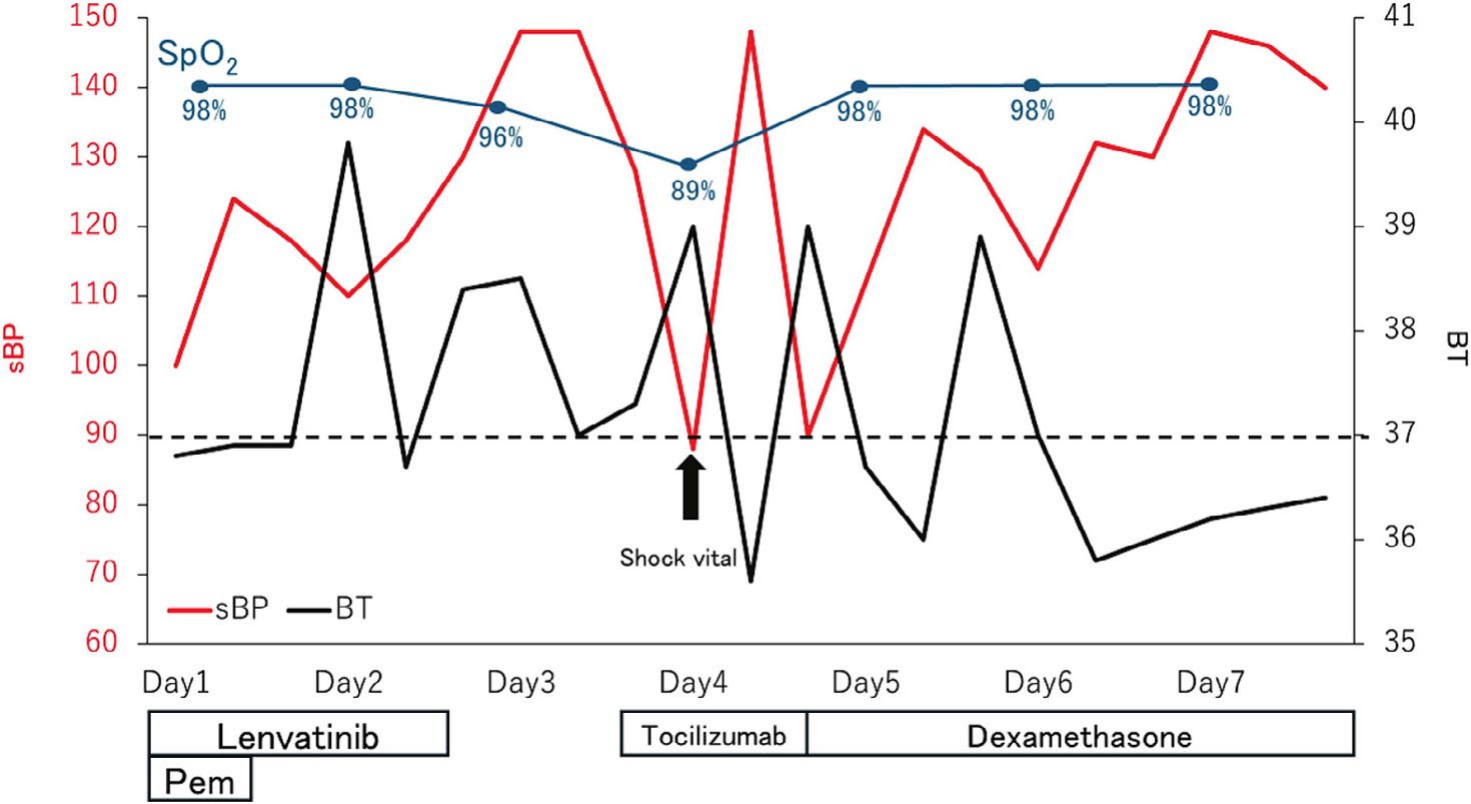
The patient developed high‐grade fever and progressive hypotension following the initiation of Lenvatinib and Pembrolizumab. On Day 4, the patient's condition deteriorated into a “shock vital” state, characterized by a systolic blood pressure (sBP) drop to 86 mmHg and SpO_2_ of 89%. Following the administration of Tocilizumab and Dexamethasone on Day 4, systemic stabilization was achieved. By Day 5, SpO_2_ recovered to 98% and sBP stabilized, despite transient fluctuations in body temperature.

A 79‐year‐old male presented with a one‐month history of low back pain and gait disturbance. Computed tomography (CT) revealed a mass in the upper pole of the right kidney and a destructive lesion in the L2 vertebra (Figure 1a,b). Suspecting metastatic bone disease, the patient was referred to our department. Although imaging was inconclusive regarding the origin of the spinal lesion, the patient underwent surgical resection and thoracolumbar spinal fusion at the orthopedic department. Histopathology confirmed metastatic clear cell renal cell carcinoma (ccRCC). Subsequently, we performed a retroperitoneal laparoscopic right nephrectomy to remove the primary tumor; the final diagnosis was ccRCC (cT3aN0M1) (Figure 1c,d). Lenvatinib plus pembrolizumab was initiated as systemic therapy. On day 2, the patient developed a fever; however, an infectious workup was negative. By day 4, he progressed to hypotension (systolic blood pressure: 86 mmHg), sinus tachycardia (110 bpm), and hypoxia (SpO_2_: 89% on room air). Laboratory findings showed elevated inflammatory markers and transaminases: CRP 22.4 mg/dL, AST 83 IU/mL, and ALT 79 IU/mL. Screening for other immune‐related adverse events (irAEs), including pituitary and adrenal insufficiency, was unremarkable (Table 1). Chest and abdominal CT revealed no acute pathologies. Based on these findings, CRS was suspected. Interleukin‐6 (IL‐6) levels were 127 pg/mL, confirming a diagnosis of Grade 2 CRS (Table 2). Tocilizumab (8 mg/kg) was administered on day 4, followed by dexamethasone (0.5 mg/kg) on day 5. The patient became afebrile by day 6, with normalization of hemodynamics and oxygenation. CRP improved to 2.41 mg/dL by day 7. Dexamethasone was gradually tapered, and the patient was transferred on day 15 (Figure 2). Following discharge, we determined that re‐challenge with either lenvatinib or pembrolizumab should be avoided to prevent the recurrence of CRS. Consequently, cabozantinib was initiated as second‐line systemic therapy. However, after 5 months of treatment, the patient developed progressive disease (PD). Axitinib was then administered as third‐line therapy; however, the patient experienced further tumor progression after 7 months and ultimately died of disease. Notably, no recurrence or symptoms suggestive of CRS were observed throughout the courses of these subsequent tyrosine kinase inhibitor therapies.

**TABLE 1:** Laboratory testing showed no findings strongly suggestive of immune‐related adverse events (irAEs).

	Day 2	Day 4	Day 5	Day 6	Day 7	Day 10
WBC (10*3/μL)	3.8	3.4	3.6	5.1	4.9	4.4
Hb (g/dL)	9.9	9.3	9.0	8.9	8.8	9.9
Plt (10*3/μL)	105	89	85	106	139	164
AST (U/L)	76	83	93	58	46	—
ALT (U/L)	63	79	88	73	79	—
LDH (U/L)	282	270	295	267	245	—
ALP (U/L)	545	613	1031	859	589	—
γ‐GTP (U/L)	255	253	331	294	271	—
CRP (mg/dl)	22.41	—	—	—	2.41	0.63
Ferritin (ng/mL)	—	3033	—	3049	—	1174
IL‐2R (U/mL)	—	8630	—	—	—	2631
IL‐6 (pg/mL)	—	127	—	—	—	—

**TABLE 2:** American Society for Transplantation and Cellular Therapy (ASTCT) consensus grading system for cytokine release syndrome (adapted from Lee et al., 2019).

CRS parameter	Grade 1	Grade 2	Grade 3	Grade 4
Fever	Temperature ≥ 38°C			
With either				
Hypotension	None	Not requiring vasopressors	Requiring one vasopressor with or without vasopressin	Requiring multiple vasopressors (excluding vasopressin)
And/or				
Hypoxia	None	Requiring low‐flow nasal cannula or blow‐by	Requiring high‐flow nasal cannula, facemask, nonrebreather mask, or Venturi mask	Requiring positive pressure (e.g., CPAP, BiPAP intubation and mechanical ventilation)

Abbreviations: BiPAP, bilevel positive airway pressure; CPAP, continuous positive airway pressure; CRS, cytokine release syndrome.

*Source:* Reprinted from Lee DW, etc. Biol Blood Marrow Transplant. 2019 Apr;25 (4):625–638.

## 3 Discussion

CRS is a systemic inflammatory response triggered by diverse factors, including infection and immunotherapy. It was first described in 1989 as an influenza‐like syndrome following the administration of OKT3, an anti‐CD3 monoclonal antibody used in renal transplantation [3]. While CRS is a hallmark toxicity of CAR‐T cell therapy, its incidence as an irAE from PD‐1 inhibitors remains exceedingly rare, reported at less than 0.01% [4, 5]. In this case, CRS was likely induced by pembrolizumab; lenvatinib's contribution is improbable, as no previous reports link this tyrosine kinase inhibitor to such immune‐mediated events. Given the lack of established management guidelines for ICI‐induced CRS, our therapeutic approach was adapted from established CAR‐T cell therapy protocols. Diagnosing ICI‐mediated CRS is challenging due to the significant symptomatic overlap with other irAEs and sepsis. Differentiating CRS from sepsis is particularly critical, yet notoriously difficult, as the treatments for CRS can be detrimental in the setting of infection. Notably, many patients with severe CRS meet the current Sepsis‐3 criteria, defined as suspected infection with a SOFA score increase of 2 points or more [6]. This diagnostic overlap creates a profound therapeutic dilemma: treating suspected CRS with high‐dose corticosteroids or IL‐6 inhibitors (e.g., tocilizumab) may exacerbate an underlying infection by suppressing the host immune response. Conversely, delaying CRS treatment to exclude sepsis may lead to rapid clinical deterioration. Consequently, a multidisciplinary approach and empirical broad‐spectrum antibiotics are often required until a definitive diagnosis is reached. This highlights the urgent need for refined diagnostic criteria to distinguish ICI‐mediated hyperinflammation from microbial sepsis. Future research into cytokine profiling may identify specific interleukin patterns, enable more precise interventions and improving patient safety.

## 4 Conclusion

As the clinical applications of ICIs continue to expand, the incidence of associated irAEs is increasing. Although CRS remains a rare complication, it is a critical differential diagnosis in patients presenting with fever, hypotension, and hypoxia. Early recognition and prompt intervention are essential to improving clinical outcomes in these patients.

## Ethics Statement

The authors have nothing to report.

## Consent

Written informed consent was obtained from the patient.

## Conflicts of Interest

The authors declare no conflicts of interest.

## Data Availability

The data that support the findings of this study are available on request from the corresponding author. The data are not publicly available due to privacy or ethical restrictions.
